# Air–Oxygen Blenders for Mechanical Ventilators: A Literature Review

**DOI:** 10.3390/s22062182

**Published:** 2022-03-11

**Authors:** Gabryel F. Soares, Otacílio M. Almeida, José W. M. Menezes, Sergei S. A. Kozlov, Joel J. P. C. Rodrigues

**Affiliations:** 1 Department of Electrical Engineering, Universidade Federal do Piauí, Teresina 64049-550, Brazil; gabryelfsoares@ufpi.edu.br (G.F.S.); otacilio@ufpi.edu.br (O.M.A.); 2 Departament of Telematics, Federal Institute of Ceará, Fortaleza 60040-531, Brazil; wally@ifce.edu.br; 3 Photonics and Optoinformatics Faculty, ITMO University, 191002 St. Petersburg, Russia; kozlov@mail.ifmo.ru; 4 Research, Post-Graduation, and Innovation, Senac Faculty of Ceará, Fortaleza 60160-194, Brazil; 5 Covilhã Delegation, Instituto de Telecomunicações, 6201-001 Covilhã, Portugal

**Keywords:** blender, respiratory diseases, control, Internet of Things (IoT), fraction of inspired oxygen, oxygen saturation, COVID-19

## Abstract

Respiratory diseases are one of the most common causes of death in the world and this recent COVID-19 pandemic is a key example. Problems such as infections, in general, affect many people and depending on the form of transmission they can spread throughout the world and weaken thousands of people. Two examples are severe acute respiratory syndrome and the recent coronavirus disease. These diseases have mild and severe forms, in which patients gravely affected need ventilatory support. The equipment that serves as a basis for operation of the mechanical ventilator is the air–oxygen blender, responsible for carrying out the air–oxygen mixture in the proper proportions ensuring constant supply. New blender models are described in the literature together with applications of control techniques, such as Proportional, Integrative and Derivative (PID); Fuzzy; and Adaptive. The results obtained from the literature show a significant improvement in patient care when using automatic controls instead of manual adjustment, increasing the safety and accuracy of the treatment. This study presents a deep review of the state of the art in air–oxygen benders, identifies the most relevant characteristics, performs a comparison study considering the most relevant available solutions, and identifies open research directions in the topic.

## 1. Introduction

Respiratory diseases are the main cause of death and disability in the world where external agents such as tobacco smoke, infectious microorganisms, and pollutants in the air can induce a serial of diseases, with an emphasis on tuberculosis, chronic obstructive pulmonary disease (COPD), and acute lower respiratory tract infections [1]. Infections such as pneumonia, influenza, acute bronchitis, and bronchiolitis are the most common examples of acute lower respiratory tract infections, where bronchiolitis is the biggest cause of hospital admission in children under 12 months of life [2]. In addition to existing and well-studied respiratory diseases, news emerges over the years, especially due to virus mutation, as is the case of the Severe Acute Respiratory Syndrome (SARS), in 2002; Middle East Respiratory Syndrome (MERS), in 201; and most recently, the SARS-COV-2 disease (COVID-19), in 2019 [1,3].

The first case of SARS occurred in November 2002 and rapidly spread around the world. The causative virus was initially detected in Himalayan palm civets, on a live-animal market in Guangdong, China [4]. Many studies about the real origin of the virus were performed, but still without a conclusion, so it is more likely that the original host was a bat, because bats are a reservoir of a wide variety of coronaviruses [5,6,7]. The Middle East respiratory syndrome was initially notified in 2012 when a patient transferred from Qatar to London Intensive Care Unit (ICU). This 49-year-old man developed a mild undiagnosed respiratory disease while visiting Saudi Arabia in August 2012 [8]. The relationship between sick camels and notifications of MERS was observed. After some studies, the relationship between sick camels and notifications of MERS was observed. It was concluded that camels are the intermediary agents since the virus did not remain in their bodies after some time, and bats are the real source of the virus, first infecting camels at some point in the past that, in turn, transmits to humans [6,9,10].

In December 2019, many pneumonia cases were related in Wuhan, China, where the common point between these cases was the seafood market. The causative agent until then was unknown. After a series of studies was discovered that a virus by the corona family was responsible, presenting a similarity great than 95% with bats coronavirus and great than 70% similarity with the SARS virus. So the conclusion was that the original host are bats [11,12,13].

**Figure 1:**
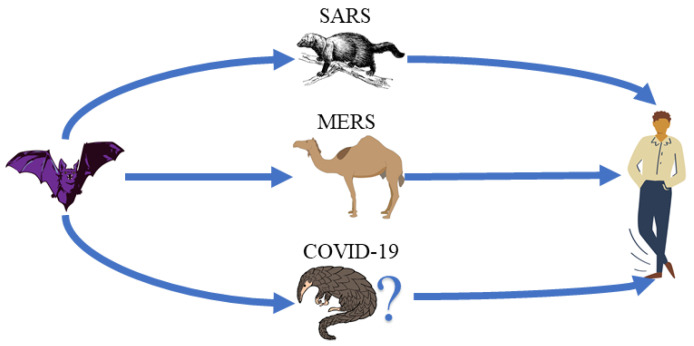
Simplified transmission scheme with likely hosts.

Studies point to the mammal Pangolin since a 91% similarity was found between the coronavirus present in species and the cause of COVID-19. Despite this percentage, results are still uncertain, requiring further studies on a topic to reach concrete conclusions [14,15,16,17]. In Figure 1, a simplified schematic of the transmission of SARS, MERS, and COVID-19 until reaching the human being is presented.

As with SARS and MERS, the most common symptoms are fever, dry cough, and tiredness, but it is possible to develop severe symptoms such as difficulty breathing or shortness of breath, pain or pressure in the chest, loss of speech or movement [18,19]. Also, it is possible for asymptomatic cases to occur, which can also infect other people since they have a high viral load in the body [20]. As may be seen in the above-mentioned examples, the treatment of many respiratory problems requires oxygen aid and mechanical ventilation, being the difference between the patient’s life and death [21]. Structural limitation of health units was evident during the COVID-19 pandemic, as there were cases of lack of mechanical ventilators and ICU beds. Patients with COVID-19 can stay up to 4 weeks using mechanical ventilation [21].

Although the vaccine against COVID-19 is being applied to the population, the contamination rates continue, especially in countries whose speed of the vaccination process is slow. Associated with this is the fact that variants of COVID-19 are emerging and presenting a higher degree of transmissibility, as is the case of the Beta (detected in May 2020), Alpha (detected in September 2020), Delta (detected in October 2020), Gamma (detected in November 2020), and Omicron (detected in November 2021) [22,23,24,25].

The use of different technologies, mainly artificial intelligence (AI) and Internet of Things (IoT), have been shown to be extremely important in combating the COVID-19 pandemic. For instance, the use of AI allows monitoring the appropriate social distance to prevent virus proliferation, supporting diagnoses from medical images of patients’ lungs, and even assessing patient’s cough if there is any indication of illness or if it is environmental [26,27,28,29,30].

It is possible making predictions based on data available to obtain an estimation of the number of contaminants over weeks. This type of study is done using linear regression techniques and artificial intelligence, as in [31]. It uses a sliding window method based on data obtained from the State Health Departments of Piauí, Rio de Janeiro, São Paulo, Santa Catarina, and Rio Grande do Sul states (in Brazil).

This paper performs a systematic review of the state of the art in the topic, searching in all the scientific databases where the topic air–oxygen blenders are addressed. The guiding question of the work is the feasibility of developing an automated air–oxygen blender, integrated in an IoT-based approach based on the most updated and promising technologies. The main objective of this paper is to carry out an in-depth review of the state of the art in air–oxygen blenders, identifying their relevant characteristics, carrying out a comparative study, and identifying open research topics on the subject. It is important to mention that published works where the main topic is “air–oxygen blender” are scarce and the vast majority are outdated since mechanical ventilation, being an important research topic (considering also this pandemic period and its importance for saving lives), is not receiving much attention in terms of published research works. Therefore, the main contributions of this paper are the following:

- a systematic review of the literature on air–oxygen blender technologies;
- a comparative analysis of the most relevant proposals available in the literature;
- the use of air–oxygen blenders in newborns;
- identification of relevant open research topics on air–oxygen blender technologies.

The remainder of this paper is organized as follows. Section 2 addresses mechanical ventilation and its components. Section 3 presents the most promising innovations related to air and oxygen blenders. Section 4 focuses on comparative analysis and discussion of the most promising technologies in oxygen blenders and Section 5 identifies open research issues on the topic. Section 6 presents the lessons learned from the study and, finally, the main conclusions.

## 2. Mechanical Ventilation

There are two application modes for treatment with mechanical ventilation: invasive and non-invasive. In invasive ventilation, patients that cannot breathe on their own need the insertion of an endotracheal tube to allow gas exchange. On the other hand, non-invasive ventilation is performed using facial or nasal masks, used in patients that have a certain degree of consciousness supporting the natural process of breathing [32].

**Figure 2:**
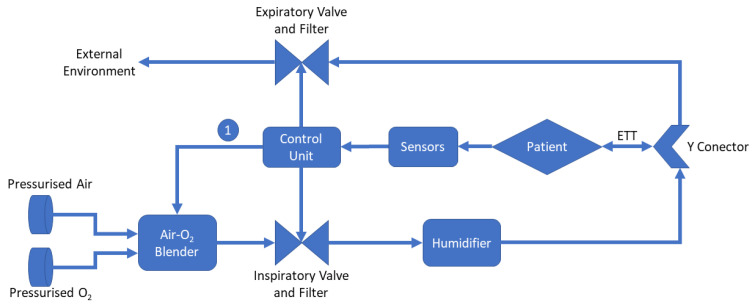
Simplified block diagram of a mechanical ventilator [36,37].

Within this equipment, a blender plays a key role for mixing air and oxygen in the proportions defined by the healthcare team, suitable for the patient’s survival. After the mixing stage, the gas is heated and humidified, then sent to the breathing circuit delivery tubes. The breathing circuit considers two pathways, one for the inspiratory phase and the other for the expiratory phase [33,34,35]. Figure 2 shows a simplified diagram of a mechanical ventilator.

The description of the blocks shown in Figure 2 are described as follows:

- Air-O_2_ Blender: Equipment responsible for mixing air and oxygen according to the healthcare professional’s settings. It is important to highlight that depending on the mechanical fan model, such adjustment can be performed automatically through the control unit, as exemplified by indicator “1” in the figure [36,37];
- Control Unit: Control unit is responsible for processing all the data in the system. From the information obtained by the sensors, it adjusts the system parameters, displays information, and, depending on the existence of automatic controls in the blender, it changes the air–oxygen ratio;
- Inspiratory and Expiratory Valve: Valves are responsible for ensuring that gas flow only goes in one direction covering the entire breathing circuit. In addition, filters are present to avoid contamination or any other risks [36,37];
- Humidifier: Humidifier is essential to avoid cases of hypothermia, rupture of the airway epithelium, bronchospasm, atelectasis, and airway obstruction [38];
- Y Conector: The Y-connector is used to join the inspiratory tube with the expiratory tube;
- Endotracheal Tube (ETT): It is a tube placed between the vocal cords through the trachea enabling oxygen delivering, inhaled gases to the lungs, and protecting the lungs from contamination [39].

Despite saving lives, using invasive mechanical ventilation can damage seriously patients’ health. This may be related to the direct mechanical effects of an intrathoracic pressures generated by mechanical ventilation. Studies show that using mechanical ventilation for at least one week is associated with important consequences in long-term physical, cognitive, and mental health of patients when they leave an intensive care unit (ICU) [21,32,40,41]. According to the literature review performed by [42] the mean of fatal cases with the use of invasive mechanical ventilation, in the best case, was 43%, while in the worst case it reached 64%. Stratifying by region, Asia had a case fatality rate of 45%, the Middle East an average of 38%, Europe 40%, North America 61%, and South America 72%.

Mechanical ventilation with automated adjustment provides significant improvements in patients’ quality of life. On the other hand, the costs of production, research, and development of equipment increased significantly, making them less affordable for less developed countries. To ensure the best use of mechanical ventilation, it should be borne in mind that it is not enough just to have an equipment, it is also necessary the adequate training of the teams, the infrastructure of the health unit, and the appropriate medications [43].

## 3. Main Technologies in Air–Oxygen Blenders

**Figure 3:**
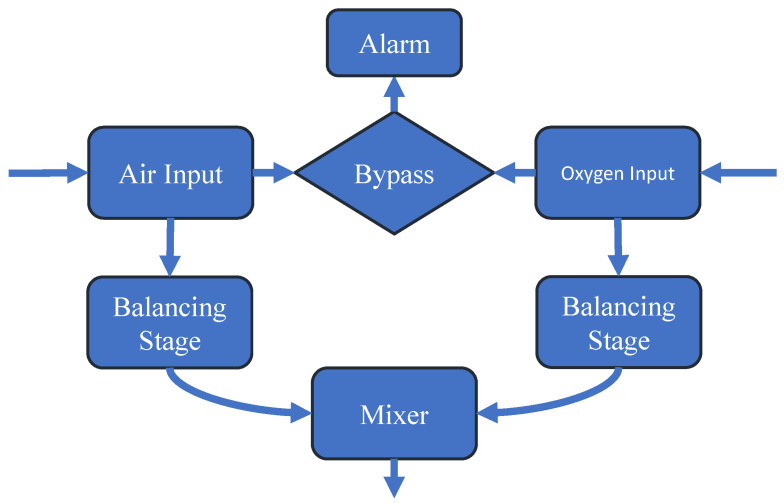
Simplified block diagram of the operation of an air–oxygen blender.

A mechanical ventilator used in the treatment of COVID-19 has a crucial component, an oxygen blender. It is responsible for providing an adequate mixture of oxygen and air to a patient in each breath, the so-called fraction of inspired oxygen (${\mathrm{FiO}}_{2}$), where a recommendation for a person is from 21% up to 100% of ${\mathrm{FiO}}_{2}$ (Fraction of inspired Oxygen) [44,45,46]. A simplified block diagram of the operation of an air–oxygen blender is shown in Figure 3.

Each block presented in Figure 3 corresponds to a stage of the equipment operation [47,48,49,50]. The description is given as follows:

- Air Input and Oxygen Input: At the entry stage of each gas, there are filters to prevent contamination and one-way valves to ensure the gas flow does not return;
- Bypass and Alarm: The bypass system checks the differential pressure between both gases. If this value is higher than the established limit, a command is sent to the alarm in order to provide an audible indication that something is wrong. In addition, the gas flow is diverted all the way to the system outlet, thus ignoring the balancing stage;
- Balancing Stage: The balancing stage considers two chambers in sequence. At this point, the pressure of the two gases is equalized in order to ensure that, at the mixing stage, the pressures are equal, always limited by the lowest pressure value between the two gases;
- Mixer: The mixing of gases is usually carried out through a proportional valve. This mechanism is based on an adjustment made by the equipment operator where the percentage of oxygen is defined. The higher percentage of oxygen desired, the smaller the airflow will be and the greater the oxygen flow at the mixing point. The equipment manufactured commonly has this control occurring manually; however, it is possible to find works with application of automatic controllers. It is important to mention that in the structure of an air–oxygen blender there is also a presence of flow and pressure sensors to inform operators how the device is working.

Oxygen treatment should be carried out with great care since depending on the amount administered to patients and time of exposure, treatment can cause series of problems such as a lung injury [51,52]. An adequate dosage depends on patient parameters monitoring, such as arterial blood gases and oxygen saturation level. Current blenders work with a manual adjustment, which is an imprecise and very general way, and it can sometimes happen that a patient receives a wrong dosage for not having the most common symptoms [46,53,54].

### 3.1. Types of Blenders

**Figure 4:**
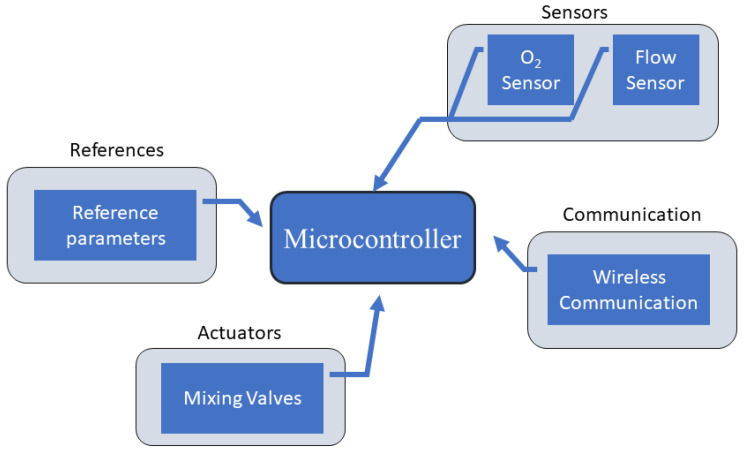
A block diagram of the system proposed by [54].

Aiming to solve the problem of adjustment in air–oxygen blenders, an auto-controlled air–oxygen blender was created by [54]. This system adjusts the percentage of oxygen in the mixture automatically according to the readings of installed sensors, mainly the flow and oximetry, adjusting the electronic valves through CPU commands. Thus, this system always supplies a patient according to his needs. The author also highlights that it is possible to receive the collected data through wireless communications. The block diagram of the proposed system is shown in Figure 4.

An air–oxygen blender at ambient pressure is a device capable of mixing air and oxygen without the need for high-pressure air. The main advantage of this type of devices is the possibility for being used to treat patients at home without the need to move to an hospital [55,56].

The prototype presented by [55,56] has a key element, variation of the poppet-seat configuration area, where oxygen and air inlet valves have different diameters. When negative pressure is induced at the blender’s output terminal, the difference in diameter becomes fundamental, since from that point on the liquid force between the two inlet valves remains unbalanced. This results in an increase of the gas passage area on one side, which is a proportional adjustment to the mixture flow required by the fan. Results obtained showed that it was possible to perform a flow variation from 5 L/min to 160 L/min, with a maximum error rate of 4% regarding the desired ${\mathrm{FiO}}_{2}$ value.

Another type of ambient pressure air–oxygen blender is based on a Venturi tube where unlike a blender valve, it is no need to have the gases at high pressure or electricity to work. The system consists of a Venturi tube with an opening for the entry of air in the middle, oxygen gas enters on one side while the air–oxygen mixture exits on the other side of the tube. Oxygen passes through the tube until it reaches the narrowest part where the air inlet window starts, so according to the Venturi effect, there is an acceleration in the gas flow while reducing the pressure in the air window. Thus, the difference between ambient pressure and that of the air intake window causes the air to be drawn into the tube, mixing with oxygen and generating the mixture at the end [57,58].

The adjustment of the mixture is carried out by changing geometric parameters of the tube, such as the diameter of the narrowest part, size of the air window, and diameter of the inlet hole after the window. In the experiments performed by the authors, a range of 34% to 100% ${\mathrm{FiO}}_{2}$ was obtained, with a flow rate of 1 L/min up to 15 L/min [58].

The blender proposed by [59] includes a proportional solenoid valve with three terminals, two for input and one for output. Measurements are made using a pulse oximeter connected to a patient to obtain a percentage of blood oxygen saturation (${\mathrm{SpO}}_{2}$). With this information, the system calculates the percentage of ${\mathrm{FiO}}_{2}$ that must be delivered to the patient using a microprocessor. This reference value is configured in a digital adaptive controller, thus adjusting the valves automatically. The range of ${\mathrm{FiO}}_{2}$ that can be delivered by this system is from 21% up to 100%.

Another automatic system is described by [60], whose main characteristics are the acquisition of patient data through sensors, the processing of this information in order to adjust the percentage of ${\mathrm{FiO}}_{2}$ to be delivered, and the flow control. If the data obtained by sensors show any type of error or interference, the processor discards that information and maintains a predefined ${\mathrm{FiO}}_{2}$ value.

The control system detects when there is a drop in pressure at the air inlet so that when it falls below an indicated limit, a compressor is automatically activated in order to normalize the pressure. If the compressor does not operate, the blender circumvents the problem by allowing 100% oxygen to pass, then open a solenoid valve that supplies the oxygen pressure to the air inlet, thus regulating the pressure at the air inlet at the same time as maintains the flow required by a patient. If an oxygen supply loses pressure, the blender does the same above-mentioned process, only reducing oxygen flow to 21% and increasing airflow to 100% [60].

A system created by [61] performs a gas mixture and performs its heating and humidification, together with measurements of gas temperature, humidity, and flow to the control system. Information about a patient’s ${\mathrm{SpO}}_{2}$ and heart rate is sent to an adaptive PID control, which performs calculations and adjusts parameters to change the percentage of oxygen present in the mixture delivered to a patient. In addition, a sensing system detects if a patient has low perfusion. The authors developed a blender that can be used in conjunction with any pulse oximeter simply adjusting controller settings. Communication between the adaptive control and blender actuators and sensors can be carried out with or without wires and, in case of controller failures, there is a way to manually adjust the valves [61]. To ensure user safety, oxygen level and heart rate alarms can be set by a healthcare professional but, by default, they are defined as <80 bpm and >180 bpm for heart rate and ≤21% and ≥60% for oxygen [61].

### 3.2. Control Algorithms

In addition to the electromechanical part, the control system is essential for an air–oxygen blender, since the reliability of a mixer depends on the precision with which the controller is applied. One way is through the use of Fuzzy control, as proposed by [62]. As it is a digital control, a microcontroller is used to execute a Fuzzy algorithm. Data were obtained using flow sensors and a pulse oximeter, where the ${\mathrm{SpO}}_{2}$ values read by the oximeter were passed to a microcontroller via RS-232 protocol so that it could calculate the ${\mathrm{FiO}}_{2}$ reference value. This obtained value was converted into two reference points for the controller, the first is the airflow and the second the oxygen flow. The authors aimed to compare their results using Fuzzy control with a control system based on rules seen in the literature. For this purpose, experiments were carried out with 3 patients and data were collected and analyzed. From the experiments, they concluded that Fuzzy control adjusted the lower ${\mathrm{FiO}}_{2}$ values for the patient about the control using a rule base, an average of 23.49% ${\mathrm{FiO}}_{2}$ for Fuzzy control, and 49.99% ${\mathrm{FiO}}_{2}$ for rule-based control. Thus, it was observed that Fuzzy control reduced the risk of excessive exposure to oxygen while increasing the risk of hypoxia [62]. The average results obtained are shown in the Table 1.

The control proposed by [63] aims to be applied in respiratory circuits of newborns. The ${\mathrm{SpO}}_{2}$ value measured by a pulse oximeter is used as an input to the developed algorithm. The information is initially converted from analog to digital and sent to a microprocessor. It is responsible for processing the information and calculating the value of partial arterial oxygen pressure (${\mathrm{PamO}}_{2}$), incorporating an algorithm with two techniques to detect and correct hypoxia and hyperoxia from the measurement of ${\mathrm{SpO}}_{2}$ and ${\mathrm{PamO}}_{2}$.

The algorithm starts by comparing the calculated ${\mathrm{PamO}}_{2}$ value to the lower safety limit; if it is found that ${\mathrm{PamO}}_{2}$ is below this value, an alarm and the control are triggered. If this value is higher than the limit, the algorithm then compares the ${\mathrm{SpO}}_{2}$ value with the minimum limit, in case this measured value is less than or equal to the safety margin, a control techniques are activated [63].

The first technique performs a gradual increase in ${\mathrm{FiO}}_{2}$ employing calculation steps until the ${\mathrm{SpO}}_{2}$ value remains above the limit determined as a safety margin. In sequence, the PID controller takes the place of a gradual increase in ${\mathrm{FiO}}_{2}$ to keep the ${\mathrm{SpO}}_{2}$ and ${\mathrm{PamO}}_{2}$ values in desired references, avoiding errors in steady-state and oscillations. If the ${\mathrm{SpO}}_{2}$ value becomes lower than the lower limit, the gradual increase takes the place of the PID. This prevents the PID from generating very high control signals or with very sudden changes when ${\mathrm{SpO}}_{2}$ falls, thus avoiding a harmful ${\mathrm{FiO}}_{2}$ rate to the newborn, the hyperoxia. If ${\mathrm{SpO}}_{2}$ stays within limits steadily, the algorithm returns to the beginning and stays in that loop [63].

The experiments were performed through a simulated breathing circuit. In the first experiment, hypoxia was induced with a ${\mathrm{FiO}}_{2}$ rate equal to 18% during the first 100 s, the ${\mathrm{PamO}}_{2}$ value started to fall until it was below the minimum limit of 68 mmHg, thus triggering the control. This caused an increase in ${\mathrm{FiO}}_{2}$ and ${\mathrm{PamO}}_{2}$ to 25.5% and 100 mmHg in 20 s, leaving the hypoxia condition. After 5.5 min, the system stabilized at 21% ${\mathrm{FiO}}_{2}$ and 87 mmHg of ${\mathrm{PamO}}_{2}$, without oscillations. In the second experiment, the conditions were 14% ${\mathrm{FiO}}_{2}$ as an initial hypoxia condition and 50 mmHg lower limit for ${\mathrm{PamO}}_{2}$. Similar to the first experiment, in less than 30 s, the hypoxia condition was overcome and in 5.5 min the system stabilized at safe values and without risk of hyperoxia [63].

### 3.3. Adjustment Modes

The study carried out by [64] aimed to evaluate three methods of controlling the ${\mathrm{FiO}}_{2}$ mixture in an air–oxygen blender for premature newborns. The considered methods are the following: routine manual adjustment, optimized manual adjustment, and closed-loop control developed by the authors. For that, a pulse oximeter was used to obtain information, such as ${\mathrm{SpO}}_{2}$ and newborns’ heartbeat, as well as flow sensors to evaluate the air–oxygen mixture. The experiments were performed with the help of a medical team where the acceptable range of ${\mathrm{SpO}}_{2}$ for the normal operation was defined as 87% to 96% of ${\mathrm{SpO}}_{2}$. Another definition is that, after an adjustment, it was due 180 s for a new adjustment and, if severe hypoxia occurred, the experiment would be interrupted.

The automatic control algorithm was deployed on a computer that communicated with the blender through the RS-232 protocol. Before deployment, the control algorithm went through a phase of open-loop feasibility testing and closed-loop validation to improve the software and hardware for the study. The main variables analyzed with the experiments were the percentage of time that ${\mathrm{SpO}}_{2}$ rate remained within a determined range, the number of adjustments in the value of ${\mathrm{FiO}}_{2}$ made by the medical team and control algorithm, the frequency of situations of hypoxia (<87% ${\mathrm{SpO}}_{2}$), and hyperoxia (>96% ${\mathrm{SpO}}_{2}$) per hour, and the average duration in seconds of episodes of hypoxia and hyperoxia [64].

The results obtained from this study showed that through a routine manual adjustment method there were, on average, 3 adjustments per hour; 9.3 episodes of hyperoxia per hour with an average duration of 19.2 s; 12.7 cases of hypoxia per hour with an average duration of 19 s, and 81.7% of the time, the value of ${\mathrm{SpO}}_{2}$ remained within the desired range. For the optimized manual adjustment, the results obtained were, on average, 7.7 adjustments per hour; 4 episodes of hyperoxia per hour with an average duration of 16.4 s; 8.7 cases of hypoxia per hour with an average duration of 16.4 s, and 91% of the time the value of ${\mathrm{SpO}}_{2}$ remained within the desired range. Finally, the average values for the closed-loop automatic control method were 0.3 adjustments per hour; 4.7 episodes of hyperoxia per hour with an average duration of 10.1 s; 9.3 cases of hypoxia per hour with an average duration of 12.4 s, and 90.5% of the time, the value of ${\mathrm{SpO}}_{2}$ remained within the desired range [64]. Results obtained are summarized in Table 2. Then, there was a reduction of approximately 50% in the cases of hyperoxia and 26% of the cases of hypoxia when comparing data of the routine manual adjustment with automatic control, besides a reduction in the duration of hypoxia and hyperoxia cases, and an increase within the recommended ${\mathrm{SpO}}_{2}$ range [64].

The work developed by [65] aimed to compare four adjustment modes of ${\mathrm{FiO}}_{2}$ described in the literature, being manual adjustment and three algorithms for automatic control and regulation of ${\mathrm{SpO}}_{2}$ for low birth weight newborns, namely, machine status, adaptive control, and PID control.

The control by state machine had as input parameters the error obtained (${\mathrm{SpO}}_{2}$ minus ${\mathrm{SpO}}_{2}$ measured), its speed, and acceleration. The used PID followed the classic model, where the proportional, integrative, and derivative parameters were previously calculated and a fine adjustment was made to these parameters. Finally, adaptive control was used based on the non-linearity of the relationship between ${\mathrm{FiO}}_{2}$ and ${\mathrm{SpO}}_{2}$, thus ensuring an adjustment closer to the appropriate one [65].

The main parameter used to assess the accuracy of the control methods was the percentage of time that control maintained the output close to the reference of ${\mathrm{SpO}}_{2}$. The number of adjustments made was measured to be aware of the reduction provided to the nursing team. Sixteen experiments were carried out with seven low birth weight newborns in order to identify which control method had the best response [65].

The values of 92%, 93%, and 94% of ${\mathrm{SpO}}_{2}$ were defined as references for automatic controls while manual adjustment had no defined reference for following the hospital procedure, which was used to maintain the rate of ${\mathrm{SpO}}_{2}$ between 90% and 96%. The authors analyzed the percentage of time that value of ${\mathrm{SpO}}_{2}$ remained exactly equal to the reference and within a variable error range of ±1–±5% regarding the desired. For error rate of ±5%, the adaptive control maintained the ${\mathrm{SpO}}_{2}$ range about 90% of the time, PID preserved the desired value approximately along 86% of time, and for state machine control the percentage was approximately 88%. In all situations, the adaptive control presented the best response, reaching approximately 20% of the total time of the experiments in the exact value of the used reference, while the manual adjustment was only 10% of the time. PID control and state machine control had similar results in this regard, being close to adaptive control [65].

Regarding the adjustment in the ${\mathrm{FiO}}_{2}$ parameter performed per hour, adaptive control presented a rate of 0.2, while the PID stayed with 0.45 and the control per state machine about 0.48. The control by manual adjustment obtained a count of 3.74 adjustments per hour with the highest rate being triggered. The total hours of experiments for adaptive control, PID, state machine, and manual adjustment were 21.42, 42.2, 14.72, and 18.43 h, respectively. The results obtained were compiled in Table 3. Thus, after completing the experiments, it was possible to conclude that among the methods evaluated in this work, adaptive control presents the best responses for the used parameters. PID and control by a state machine presents a very similar performance [65].

### 3.4. Use of Blenders in Newborns Treatment

The air–oxygen mixer is a versatile and fundamental component of mechanical ventilators being used in both adult and pediatric patients. However, due to the different physiological characteristics of these groups of patients, the blender adjustment performed by the medical team must follow appropriate parameters to avoid risks [66].

Among the characteristics that may turn newborns needing respiratory assistance are weakness of the respiratory muscles, immaturity of the rib cage, and surfactant deficiency. The risks for newborns are most susceptible when treated with inadequate oxygen therapy are blindness from retinal maldevelopment, lung damage, brain damage from hyperoxia, and hemolytic anemia. Although there is no exact value of ${\mathrm{FiO}}_{2}$, there is a consensus that this value should initially be about 30% and adjusted according to the variation of ${\mathrm{SpO}}_{2}$, since initial values below 30% of ${\mathrm{FiO}}_{2}$ generate significant risk of hypoxia [67,68,69]. Because they are so vulnerable, a series of factors can interfere with the increase in the rates of ${\mathrm{SpO}}_{2}$ in newborns, making it necessary for the health team to be always warned to the procedures to be followed, the characteristics of each patient, the parameters of the equipment used, and environmental conditions [67,68].

In a global context, the use of air–oxygen blends for newborns treatment is quite common in more developed countries, but the same cannot be mentioned for developing or low-income countries. In these regions, the acquisition of blenders for being used in health units comes up against financial issues, given the high price of equipment, which is usually important in developed countries that have the domain of technologies [66]. An example is presented by [70], which shows that only 1% of health facilities in Sub-Saharan African countries have air–oxygen blenders, so it is common to administer oxygen without precise control, being monitored only intermittently and limitedly.

## 4. Comparative Analysis and Discussion

From the analyzed studies, it was possible to notice the differences between a conventional air–oxygen blender, which uses both oxygen gas and air (both pressurized) and an air–oxygen blender that does not use pressurized air, described in the literature for residential applications or in places with few financial resources.

The two ways described for blending with air at ambient pressure related to the use of a Venturi tube and poppet-seat valves. The use of a Venturi tube to perform an air–oxygen mixture has some disadvantages. As seen, this type of blender is more inaccurate, since the adjustment is made manually, directly changing the size of the air inlet window and the diameter of the through-holes of oxygen gas and mixture, something that is done on a scale millimeter. Furthermore, depending on the combination of the sizes of moving parts, different results can be obtained. Finally, the mixing flow that can pass in a range of 1 L/min to 15 L/min. Despite disadvantages, which end up making its use unfeasible in hospital environments, it is a technology that can be successfully applied in regions with less economic resources or cases that do not require significant changes in the rates of ${\mathrm{FiO}}_{2}$ delivered to the patient.

The use of a blender with poppet-seat valves has a mixture flow rate ranging from 5 L/min to 160 L/min, which already represents a significant improvement over the use of a Venturi tube. In addition, due to the constructive configuration of a blender, as one of the valves is pressed, the other is pushed proportionally, thus ensuring a correct supply of ${\mathrm{FiO}}_{2}$. However, as with the use of Venturi, one of the limitations is that this type of blender is not suitable for hospital usage, being restricted to residential applications in cases not so severe. A summary comparison between these two types of blender is shown in the Table 4.

Regarding conventional blenders, the technological improvements can be achieved by using different types of sensors to increase the accuracy, wireless data transfer to enable implementation in a distributed context, automatic control algorithms to improve overall system performance. As presented in several studies, performing manual adjustments on blenders is very imprecise and can cause serious consequences for patients, mainly because the relationship between ${\mathrm{FiO}}_{2}$ and ${\mathrm{SpO}}_{2}$ has a non-linear characteristic and varies from person to person. Thus, the ideal condition for manual adjustment would be an almost constant presence of a health professional to make such adjustments as soon as they are needed but this ends up being impracticable. Table 5 contains a summary of the techniques presented in the studies discussed at Section 3.3.

The use of an automatic control system ensured the precision in the supply of an air–oxygen mixture and an adjustment was performed at the slightest sign of variation in patients’ parameters during the experiments. The studies presented in Section 3 showed that among the control techniques already used, Fuzzy control and adaptive control stand out for presenting the best efficacy and safety for patients. Despite this, some authors reported resistance of health professionals with this innovation, mainly claiming that there would be an increase in their workload or they would not trust an automatic blender. These reasons proved to be unfounded since workload for health teams tends to be reduced as technology assists their activities and also guarantees greater precision and safety for patients, adapting to each organism and its particularities.

The absence of work with applications of IoT and artificial intelligence in air–oxygen blenders was observed. Studies addressing these two topics applied to different areas of medicine are increasingly recurrent aiming mainly to assist in diagnosis of some human diseases and physical conditions.

Technology evolution provides greater integration and the evolution of modern medicine depends on the adequacy of equipment already consolidated with new technologies, always aiming to improve the population quality of life.

## 5. Open Issues

Based on the above-presented study and the performed analysis, the following open research issues in the topic are identified:

- Use of open source embedded systems: Open source platforms can significantly reduce the costs for creating and developing automated blenders due to the popularity of such platforms and the simplified implementation of the algorithms, while ensuring information security using protocols already established in the literature;
- The application of IoT in blenders and cloud data storage: a remote monitoring system for several blenders can be developed. Then, health professionals, even at distance, can monitoring patients evolution and send commands to blenders for making needed adjustments. All data generated is stored in the cloud, making all the clinic patients registries saved and easily available when needed;
- Artificial intelligence and optimization in blenders: in conjunction with IoT, it would be possible to obtain a device with extremely high precision and the ability to adapt to unforeseen events that may occur, since the machine itself would detect any anomalies and immediately communicate to the health staff when making adjustments for return the patient to normal condition. It was also observed the possibility of reducing the production costs of a blender from the use of 3D printing, which can facilitate the access to this important equipment;
- Development of modular blenders: The development of modular equipment is an area that can add positively to hospital electronics. This would allow for easy integration of an external blender into a mechanical fan through standardized protocols. In addition, it would only allow the removal and replacement of the blender for periodic maintenance without compromising the use of the mechanical fan. Modular devices tend to be more versatile and more cost-effective.

## 6. Lessons Learned and Conclusions

It should be noted that blenders are a crucial component of the mechanical fan, being the equipment that does most of the work in the ventilation process. Even so, it stills little explored in the literature, hardly being treated as a central and prominent piece. Innovations for such equipment end up turning to control algorithms, which, in practice, are not yet so widespread in commercially available blenders.

From the performed literature review, it was possible to observe the need to create air–oxygen blenders with automatic control. The improvements provided by this type of equipment in blenders with a manual adjustment range from the precision in the rate of ${\mathrm{FiO}}_{2}$ provided, the guarantee of the ${\mathrm{SpO}}_{2}$ value to follow the proposed reference (as much as possible) until patient safety is assured since as it is an automatic system, monitoring occurs 24 h a day always being adapted according to the patients react to treatments. The workload in health professionals tends to decrease as well as cases of hypoxia and hyperoxia. The main factor that makes implementation difficult is the associated cost, which for many companies becomes unfeasible.

An alternative would be based in research investments using open-source embedded platforms always working with the idea of modular equipment. The use of embedded systems, such as Arduino, ESP, and Raspberry Pi, together with 3D printing techniques that are increasingly popular, can provide economically and technically viable solutions for the development of low-cost blenders aimed especially at developing/lower countries. an automatic adjustment of parameters can be performed using control techniques already established in the literature, such as Fuzzy-based approaches.

Another point worth mentioning is the connectivity of this equipment using an IoT-based approach. The best use of this technologies can generate very positive results since it allows information sharing among health professionals and teams more efficiently. The patients’ monitoring can be performed in real-time, at distance by the medical staff, allowing also communication between the more diverse devices of a hospital wing. To follow this approach, it is also necessary invest in information security. As it is a vital piece of equipment, all the parameters and information exchanged from/to devices must be protected with strong encryption and other security policies already established in secure applications. The automated blender itself cannot achieve its maximum performance without a foundation of infrastructure and human training behind it. All the information must be kept and protected.

## Figures and Tables

**Table 1:** Average results for Fuzzy and Rule-based controllers studied in [62].

	${\mathrm{FiO}}_{2}$— Fuzzy Control	${\mathrm{FiO}}_{2}$—Rule-Based Control
Maximum	60.80%	100%
Average	23.49%	49.99%
Minimum	21%	21%

**Table 2:** Results obtained from the work of [64].

Parameter	Routine Manual Adjustment	Optimized Manual Adjustment	Closed-Loop Automatic Control
Adjustments per hour	3	7.7	0.3
Episodes of hyperoxia per hour	9.3	4	4.7
Average duration of hyperoxia episodes (seconds)	19.2	16.4	10.1
Cases of hypoxia per hour	12.7	8.7	9.3
Average duration of hypoxia cases (seconds)	19	16.4	12.4
Percentage of time that the ${\mathrm{SpO}}_{2}$ value remained as desired	81.7%	91%	90.5%

**Table 3:** Summary of results obtained in [65].

Type of Control	Total Hours	Total Manual Adjustments	${\mathrm{FiO}}_{2}$ Adjustments per Hour
Adaptive control	21.42	5	0.2
PID	42.2	19	0.45
State machine	14.72	7	0.48
Manual adjustment	18.43	69	3.74

**Table 4:** Comparison between blender with Venturi Tube and Poppet-seat Valves.

	Venturi Tube	Poppet-Seat Valves
Range of mixing volume obtained	1 L/min to 15 L/min	5 L/min to 160 L/min
Main Advantage	It is a technology that can be successfully applied in regions with less economic resources or cases that do not require significant changes in the rates of ${\mathrm{FiO}}_{2}$ delivered to the patient	Capable of providing greater mixing flow and greater precision compared to the Venturi tube model.
Main Disadvantage	This type of blender is more inaccurate, in addition to not being able to be applied in the hospital environment, only residential.	Not suitable for hospital use.

**Table 5:** Comparative between the adjustment of presented techniques.

	${\mathrm{FiO}}_{2}$ Adjustments per Hour	Percentage of Time That the ${\mathrm{SpO}}_{2}$ Value Remained as Desired
Routine manual adjustment	3	81.7%
Optimized manual adjustment	7.7	91%
Closed-loop automatic control	0.3	90.5%
Adaptive control	0.2	90%
PID	0.45	≈86%
State machine	0.48	≈88%

## Data Availability

Not applicable.
